# Cytocompatibility assessment of an anaerobic adhesive for use in implant dentistry: An in vitro study

**DOI:** 10.34172/japid.026.4208

**Published:** 2026-05-13

**Authors:** Barbara Magalhães Figueiredo Dias, Clara Almeida Mares, Beatriz Gomes de Lucardians, Talyta Couto de Freitas, Ana Flor Sá, Danilo Rocha Dias, Ivana Márcia Alves Diniz, Frederico Santos Lages

**Affiliations:** ^1^School of Dentistry, Universidade Federal de Minas Gerais, Belo Horizonte, Brazil; ^2^Department of Restorative Dentistry, School of Dentistry, Universidade Federal de Minas Gerais, Belo Horizonte, Brazil

**Keywords:** Biomechanics, Cell migration assay, Cell survival, Cell viability, Dental implants

## Abstract

**Introduction:**

This study evaluated the cytotoxic effects of anaerobic adhesive and its impact on cell viability and migration of epithelial and mesenchymal cells.

**Methods:**

The assays were conducted on commercially obtained eukaryotic cells: human keratinocytes (HaCaT) and mouse fibroblasts (L929). For cytotoxicity assessment, HaCaT cells were exposed to the adhesive extravasation after screwing the abutment‒implant assembly, and L929 cells were placed in contact with medium conditioned with the adhesive at concentrations of 0.5% and 1.0%. Cell viability was measured using the MTT reduction test, and migration was assessed with the scratch assay in L929 cells. Relative cytotoxic potential was determined with a negative control set at 100% viability. ANOVA followed by post hoc Tukey analysis was performed.

**Results:**

The evaluated anaerobic adhesive exhibited cytotoxic potential, especially in L929 fibroblasts. Cell migration was significantly reduced in mouse fibroblasts.

**Conclusion:**

This study indicated that although the application of anaerobic adhesive is limited to the threads of abutment screws and is handled responsibly, further studies are needed before recommending its use in implantology due to its cytotoxicity.

## Graphical Abstract

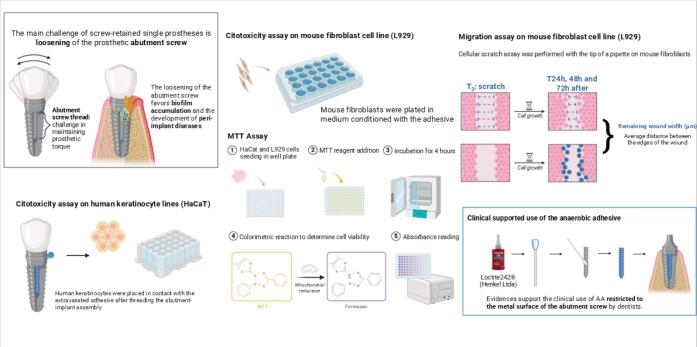

## Introduction

 Implant-supported rehabilitation is a well-established clinical practice for replacing single and multiple teeth, offering predictable outcomes and long-term success. Implant dentistry is an evolving science that incorporates multidisciplinary technologies, expanding the possibilities of surface treatments, manufacturing materials, torque capacity, reducing microgaps at the abutment-implant interface, and shortening treatment time.^1,2^

 Failure to maintain the torque achieved during prosthetic installation is clinically reflected in abutment screws loosening, which, in addition to the mechanical challenge, also facilitates microbial colonization by infectious agents related to peri-implant diseases at the abutment‒implant interface.^2-5^ Loosening of implant crowns is the most common technical complication, especially in single implants, with a cumulative incidence reported in the literature ranging from 8.8% to 16.1%.^6,7^ Factors that predispose to torque loss include inadequate preload, unbalanced occlusion, unsatisfactory prosthetic profile, variations in the implant prosthetic platform, and insufficient anti-rotational features.^8^

 The preload is the tension force generated in the prosthetic abutment screw during prosthesis installation. The stress on this component ensures locking through mechanical friction between the screw’s external surfaces and the implant’s internal surface.^8^ Another key aspect is the anti-rotational properties of implant systems, which are the focus of research aimed at fulfilling clinical needs by developing fitting systems that support the longevity of torque in more than 20 different types of connections documented in the literature.^9^ The preload and anti-rotational features are essential in preventing the prosthetic abutment screw from loosening and could benefit from chemical means that extend the counter-torque of the components.

 Alongside implant dentistry, engineering also works with screwable metal surfaces and uses adhesive materials that promote a chemical lock at these interfaces. These materials are called anaerobic monocomponent adhesives (AA) and seal the microgaps between threads, promoting locking, preventing the metal assembly from unscrewing, and thereby improving overall stability.^10,11^ Several materials have already been tested in relation to increasing counter-torque in components on dental implants, such as chlorhexidine digluconate, Vaseline, silicone gel, polytetrafluoroethylene (PTFE) tape, blood, fluoride, mouthwash, saline solution, and Loctite® adhesives 277, 243, 242 and 2400.^12^ However, still no dental material developed exclusively for this purpose is available, and those that have shown the best in vitro results are those used in engineering.

 Lyra et al.^10^ demonstrated in vitro that Loctite® 242 torque AA effectively locks the abutment‒implant assembly by increasing the counter-torque in titanium implants without damaging the screw threads. Despite these positive results and its promising clinical potential, the authors highlighted the need for further experimental studies to investigate the cytotoxicity and biocompatibility of AA.^10^ Given the recurring issue of prosthetic abutment screw loosening and the need for evidence regarding the biocompatibility of the Loctite® 242 in cellular environments, this study aimed to evaluate the biological properties of this specific adhesive through cytotoxicity, viability, and cell migration tests.

## Methods

 We used immortalized epithelial cells (human keratinocytes, HaCaT) and mesenchymal cells (mouse fibroblasts, L929) obtained commercially. The cells were expanded in complete culture media, consisting of high-glucose Dulbecco’s Modified Eagle Medium: F12 (DMEM), supplemented with 15% fetal bovine serum, 100.000 IU/mL penicillin, and streptomycin (Gibco, New York, USA), and incubated at 37ºC in a humid atmosphere containing 5% CO_2_. The culture medium was changed every 2 days, and the cells were subcultured until the research protocol was applied. Bionnovation® conical connection implants, measuring 4 × 10 mm, with their respective abutments of the same brand and model Tiprep 4.5 mm, along with the corresponding screws to stabilize the set, were used with AA Loctite® 242.^13^

###  Experimental Design

 The cytocompatibility of the adhesive was assessed in two ways: its residual effect on epithelial cells (HaCat) after screwing the implant with 1 drop of the adhesive component (10 µL), and in two dilutions (0.5% and 1.0% w/v) in indirect contact with mesenchymal cells (L929).

###  AA Challenge on Human Keratinocyte Line (HaCaT) 

 Cells were plated in 24-well microplates in quadruplicate, at a density of 1 × 10^5^ cells/well. The abutment‒implant set from the test group was prepared by coating the entire length of its abutment screw threads passively with the equivalent of one drop of AA (10 µL) and incubating it in 1 mL of complete media. As seen in other studies,^10,11^ one drop of adhesive is sufficient to prevent excess and to fill all internal turns of the prosthetic screw. The control group was prepared by incubating one abutment‒implant set that received no AA on its abutment screw. Following the manufacturer’s recommendations, all implants were torqued to 20 Ncm. Samples were incubated in a humidified atmosphere containing 5% CO_2_ at 37°C. The cells were analyzed after 12 and 24 hours using the MTT reduction assay.

###  AA Challenge on Mouse Fibroblast Cell Line (L929)

 Cells at a density of 1 × 10^5^ cells/well were plated in 24-well microplates in quadruplicate. A control group with complete culture media and two test groups with conditioned media were assessed. The test media were conditioned with 0.1 g or 0.05 g/10 mL of medium, i.e., 1.0% and 0.5% w/v, respectively.^14^ Samples were incubated in a humidified atmosphere containing 5% CO_2_ at 37°C. The medium was refreshed every other day. Cell viability was analyzed at 24, 48, and 72 hours using mitochondrial activity analysis of the cells submitted to the MTT reduction assay. At the same experimental times, the cell scratch assay was used to evaluate cell migration.

###  Cell Viability Analysis

 Cell viability was assessed using the MTT or 3-[4,5-dimethyl-thiazol-2-yl]-2,5-diphenyltetrazolium bromide reduction test. This assay consists of evaluating the reduction of the water-soluble yellow tetrazolium salt, MTT, to the water-insoluble purple formazan crystals by metabolically active cells.^15-17^ The MTT solution was prepared by dissolving 0.05 g of powder in 10 mL of phosphate-buffered saline (PBS, pH = 7.2). At the time of each analysis, the MTT solution and fresh medium (1:10) were added to each well. The culture plate was incubated at 37°C in a humidified atmosphere containing 5% CO_2_ for 4 hours. The plate was then transferred to the inverted-phase microscope to confirm the formation of formazan crystals. Once the MTT solution had been removed, 150 μL of dimethyl sulfoxide (DMSO, Sigma-Aldrich, San Luis, Missouri, United States) was added to each well. A blank group consisting of pure DMSO was used for MTT normalization. The purplish-blue formazan was solubilized, and its absorbance was determined by optical density in a Spectra Max 190® spectrophotometer (BioTeK®, Instruments, Inc., Winooski, Vermont, USA) with a 540-nm filter. The readings were taken using Soft Max® software (Molecular Devices, San Jose, CA, USA). Data were compared for relative cell viability, with the negative control group (100% viability) as the reference.

###  Cell Migration Analysis

 The L929 cells were plated in 24-well microplates at a density of 1 × 10^5^ cells/well. After reaching confluence, the cells were subjected to the “cell scratch” assay in quadruplicate. Using a p200 tip, a “wound” was simulated, and the width of the wound was measured over 24, 48, and 72 hours after contact with the 0.5% and 1.0% w/v concentrations. The medium was refreshed every other day. To this end, images were captured with a 4 × objective under a multimode microplate reader (Cytation 5 Cell Imaging, Winooski, VT, USA) and analyzed using Image J software.^18^

###  Statistical Analysis

 Statistical analysis was performed using the Statistical Package for the Social Sciences (IBM SPSS Statistics 22.0). Normality was assessed with the Shapiro-Wilk test, and homogeneity of variances was checked using Levene’s test before analysis. An ANOVA was performed, followed by post hoc Tukey tests.

## Results

**Figure 1:**
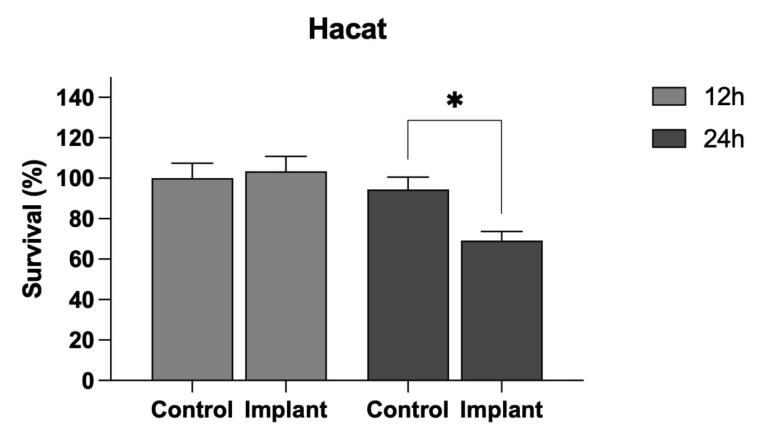
Cell viability of epithelial cells 12 and 24 hours after exposure to the anaerobic adhesive

 The cytotoxicity results (Figure 1) showed that, over the 12-hour period, there was no statistical difference in the survival rate of human epithelial cells (HaCaT) exposed to leaked AA in excess from the implant and prosthetic abutment assembly compared with the control group, which did not receive AA. Over the 24-hour period, the difference in survival between the cells was statistically significant, with a decrease in the population of human keratinocytes exposed to excess AA (approximately 70% viability).

**Figure 2:**
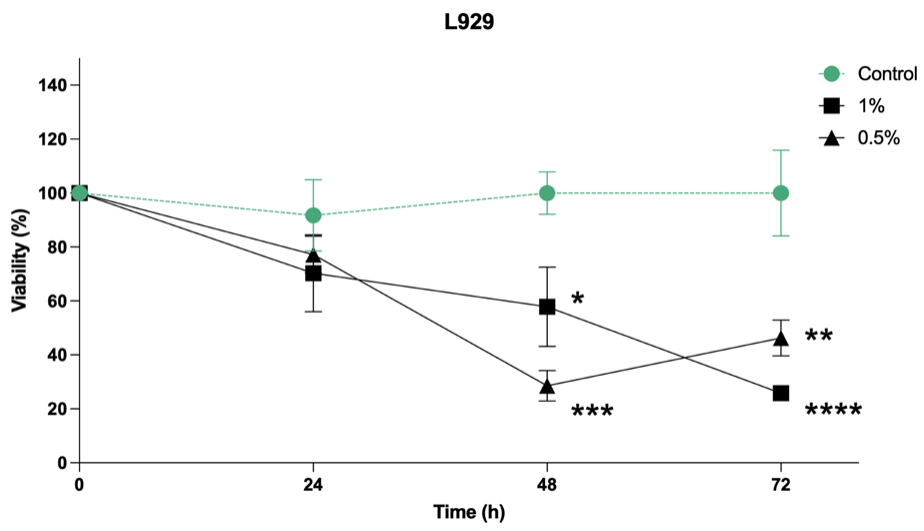
Mesenchymal cells viability at 24, 48, and 72 hours after exposure to anaerobic adhesive at concentrations of 0.5% and 1.0%

 The results of the cell viability test (Figure 2) carried out on L929 fibroblast cells showed that AA had little or no cytotoxic effect on the cell groups exposed to the material at concentrations of 0.5% and 1.0% compared to the control group in the first 24 hours (70% and 80%, respectively). Over the 48-hour period, AA at 1.0% yielded approximately 60% viability, whereas the 0.5% concentration was cytotoxic compared with the control group (viability around 30%). The 72-hour analysis showed improved cell viability in L929 cells in contact with 0.5% AA (50% viability), whereas 1.0% AA was more cytotoxic (30% viability).

**Figure 3:**
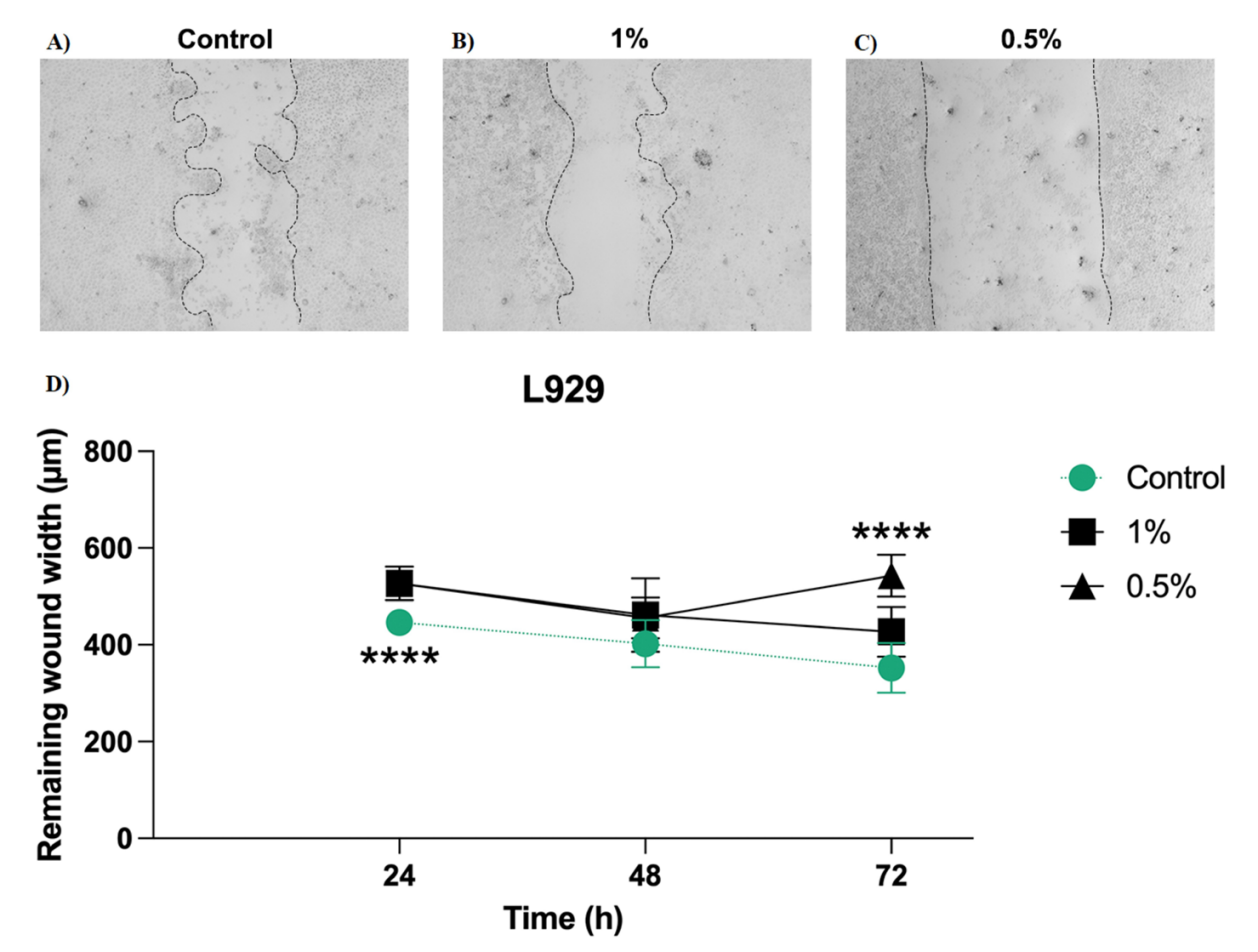
Wound healing edges in mesenchymal cells at 72h. A) control. B) 1.0%. C) 0.5%. D) 24, 48, and 72 hours after exposure to anaerobic adhesive at concentrations of 0.5% and 1.0%

 Migration analysis revealed that, after 24 hours, both groups with conditioned medium exhibited significantly less wound closure compared to the control group. By 48 hours, all the groups showed similar wound widths. At 72 hours, the 0.5% group demonstrated significantly less migration than the 1.0% and control groups (Figure 3).

## Discussion

 This study evaluated the toxicity of Loctite® 242 adhesive using cytotoxicity, cell viability, and cell migration tests, given its proven mechanical benefits. These preliminary results showed that the adhesive has cytotoxic potential, particularly in L929 fibroblasts, and that it impairs cell migration at certain time points. The data from the AA residue extravasated from the implant and prosthetic component assembly, in contact with HaCaT cells, suggest significant cytotoxic effects on epithelial cells, which were evaluated directly in contact with the AA.

 In vitro studies and clinical investigations have already demonstrated that loosening the prosthetic abutment screw is the most common failure in implant-supported rehabilitation. This issue is related to the attributes of the implant system, the type of prosthetic fixation, the force applied to the components, and their positioning in the arch.^7,19^ AA is an effective solution to this problem because it increases the counter-torque value for implants with conical and external hexagon titanium connections, without damaging the screw surface, by simply applying 1 drop of adhesive containing approximately 10 μL.^10^ Although the same effect on counter-torque was not observed for implants with a conical zirconia connection, its use made it harder to unscrew and remove the abutment, even when the screw was absent.^20^ Therefore, AA can be a valuable resource to address the issue of loosening the prosthetic abutment screw and raises the possibility that the adhesive may also be effective in sealing the abutment-implant interface.

 Cytotoxicity evaluation aims to understand how a material interacts at the molecular level with cells in culture to predict immune, genotypic, and inflammatory responses, thereby helping assess its suitability for in vivo studies and, ultimately, support its clinical use.^21^ It is essential for the safety approval of dental materials because they come into close contact with soft and hard tissues in the oral cavity, such as peri-implant structures.^22^ This analysis aimed to understand the effects of adhesive leakage applied to the prosthetic abutment screw in sufficient quantity to cover its threads during prosthetic torque, simulating the oral environment of patients.

 The cytotoxicity evaluation in this study showed that the presence of the adhesive in the cell medium for 12 hours did not cause adverse effects on keratinocytes. The survival rate only decreased after 24 hours, reaching about 70% of viable cells. A hypothesis for this result is that the late decline might be linked to the decomposition of methacrylate, a component of AA, which can induce cell apoptosis, thereby explaining the observed population reduction.^23-25^ However, the lack of targeted apoptotic assays is a limitation of the current study, and future research should address this area to verify this hypothesis.

 Similar or more severe results were observed with resin-based sealants containing calcium hydroxide, in which cell proliferation was inhibited (MTT assay) at all time intervals evaluated.^26^ Regarding endodontic ceramic biomaterials used under hard tissues, in vitro studies showed cytotoxic effects after 24 hours;^27^ 0.2% chlorhexidine mouthwash, an adjunct in the treatment of periodontal disease, led to the death of around 25% of human gingival fibroblasts in vitro;^28^ glass ionomer cements showed a cytotoxicity of ≈20% in mouse fibroblasts in vitro;^29^ dual resin cements exhibited a cytotoxicity rate of up to 90% in an in vitro study on human gingival fibroblasts;^30^ and methacrylate-based monomers used in provisional and definitive prostheses were capable of triggering hypersensitivity reactions in patients and professionals.^31,32^

 Analysis of cell viability (MTT assay) indicated mitochondrial integrity, interpreted as the cell’s ability to synthesize its energy currency.^33^ This study found evidence of deleterious effects of the adhesive on mesenchymal cells. The observed non-linear dose-response, where the 0.5% concentration exhibited higher initial toxicity than the 1.0% group, merits careful consideration. This phenomenon is likely attributed to the differential solubility and leaching kinetics of the adhesive’s hydrophobic components in a standardized volume of culture media.^25^ At the lower 0.5% w/v ratio, it is possible that a more efficient dispersion of the material may have facilitated a burst release of uncured monomers, leading to severe 48-hour cytotoxicity. In contrast, the 1.0% group may have experienced a saturation effect or particle aggregation (due to the adhesive viscous nature), delaying the peak concentration of leached toxic components until the 72-hour mark. The subsequent viability rebound observed in the 0.5% group further suggests that once the initial toxic insult stabilizes, the resilient fibroblast subpopulation can initiate a recovery phase. Although significant cytotoxicity was observed, the use of the adhesive has a favorable cost-benefit ratio because, while adhesive systems based on components similar to Loctite® 242 (methacrylate) are marketed for direct use on vital teeth, they exhibit viability below 40%. This means they are severely toxic and pose a risk of causing an irreversible inflammatory reaction, which can lead to pulp necrosis. The AA under study does not come into contact with the peri-implant tissues if handled correctly.^34-36^

 The migration test assesses the ability of cells in a monolayer to move and occupy space, similar to a closing wound, by artificially creating a gap in the monolayer with a sharp instrument, such as the tip of a pipette.^37^ The “wound” closure of this space over time demonstrates movement and indicates cell viability. In this study, the mouse fibroblasts exposed to the adhesive were less able to reapproximate the edges of the wound at 24 (0.5%) and 72 hours (1.0%) compared to the control, which may be associated with greater dissolution of the adhesive in the medium and, therefore, greater release of toxic substances into the cells. On the other hand, at 48 hours, the cells in the conditioned medium showed a proliferative capacity similar to that of the control group. Ultimately, the scratch assay results revealed a significant inhibition of cellular migration in the L929 model. This impairment may represent a critical biological limitation of the adhesive’s current formulation. In a clinical setting, the migration of fibroblasts and epithelial cells is essential for re-establishing the mucosal seal and preventing bacterial colonization.^38^ Our findings suggest that the leaching of uncured monomers likely disrupts the cytoskeletal dynamics or metabolic pathways necessary for cell motility, thereby potentially compromising the wound-healing potential of the peri-implant tissues.

 In general, while mechanical applications of the AA are promising, its interference with cellular functions suggests that any translation to in vivo models should be approached with caution in a clinical routine; considering the results of the cell viability and cell migration tests, the ideal amount for application is approximately one drop of the adhesive.^10,11,20^ The actual amount corresponds to the volume absorbed by an instrument with a fine, small tip, such as a microbrush applicator, sufficient to cover the screw threads of the prosthetic abutment without excess. After the prosthetic torque, minimal leakage is expected between the abutment and implant surfaces, which should be removed cautiously with fine-tipped instruments wrapped in gauze or absorbent cotton, preserving the integrity of the adjacent soft tissue.

 The evaluated AA is commercially available in liquid form and only initiates polymerization in an oxygen-free environment between metallic surfaces. In this context, it is expected that, after complete polymerization of the material and its internal confinement within the implant, its cytotoxicity will be reduced or will not cause clinical damage. Considering the clinical context, the oral environment itself can also delay complete polymerization at the implant‒abutment interface. Situations where there is a gap between the abutment and the implant may not provide a complete seal, allowing oxygen to enter. Temperature fluctuations associated with dietary intake can also delay complete polymerization, since the polymerization time of this AA varies with temperature.^12^

 In contrast to the tests carried out directly on living cells in this study, in dental practice, the adhesive is not recommended for direct use on the mucosa. Due to its thixotropic nature,^13^ the AA is a viscous substance that exhibits minimal run-off, enabling direct application to the threads of the screw of the prosthetic abutment outside the oral cavity, thereby decreasing the risk of contact with peri-implant tissues. The technique becomes even safer when performed with a microbrush, which follows the operator’s movements and limits the material’s contact with the metal surface of the screw.

 The results of this study serve as a baseline for necessary adjustments—such as further dilution, alternative curing methods, or chemical refinement—that are needed to improve the viability of these materials for future applications. Furthermore, it is important to note that other brands offer adhesives with similar and different formulas that share the same engineering indications. Therefore, future studies with other adhesives and formulations may provide more insights into their potential use in dentistry. The authors of this study also highlight the need for additional research to evaluate the sealing properties and durability of the counter-torque before it is used clinically. Although marginal degradation of the adhesive is not expected, since it will be confined between the implant and the prosthetic abutment screw, an investigation into potential marginal degradation resulting from the natural function of the stomatognathic system is also recommended. Additionally, the adhesive was tested on a monolayer of cells, which does not fully represent the complex environment of the oral cavity. Further testing is necessary to determine whether cellular functions might be affected by the presence of the adhesive.

 The current findings should be interpreted with awareness of several methodological limitations. As an initial study, the model does not account for the complex fluid dynamics of the oral cavity, including salivary dilution and protein adsorption, which may attenuate or modify chemical interactions in vivo. Moreover, while the 1% w/v concentration was based on established protocols for testing biomaterial dissolution products, the study lacks detailed chemical characterization and quantification of the specific components leached into the conditioned media.^14^ Future research using liquid chromatography and dynamic flow systems is essential to provide a clearer pharmacokinetic profile and confirm these preliminary findings under physiological conditions. Ultimately, using L929 and HaCat cells may not perfectly reflect human ectomesenchymal responses, and the absence of a peri-implant model limits the clinical relevance of the data. Furthermore, because this study did not include a macrophage or inflammatory response model, the adhesive’s immunotoxicological profile remains unknown.

## Conclusion

 At short time intervals, the anaerobic adhesive Loctite® 242 demonstrated cytotoxicity, affecting the viability and migration of epithelial and mesenchymal cells at both concentrations. Even if the adhesive is applied only to the threads of the abutment screws and its handling is performed responsibly, the preliminary findings of this study suggest that further studies are still necessary before recommending the use of AA in Implantology.

## Competing Interests

 The authors of this study report no conflict of interest.

## Data Availability

 Raw data will be provided upon request from the corresponding author.

## Ethical Approval

 Not applicable.
